# Knowledge and attitude of medical students on brain death and organ donation

**DOI:** 10.1186/1865-1380-7-S1-O5

**Published:** 2014-07-25

**Authors:** Elamurugan Tirthar Palanivelu, Sudarshanan Sundaramurthi, Shanmugam Dasarathan, Sreenath Gubbi Shamana, Vikram Kate

**Affiliations:** 1 Department of Surgery, Jawaharlal Institute of Postgraduate Medical Education and Research, Puducherry, India

## Objective

To assess the knowledge, attitudes of medical students regarding brain death and organ donation from brain dead.

## Methods

The study was conducted among the final year medical students of JIPMER, Puducherry using a 22-item questionnaire during the academic year 2013.

## Results

A total of 90 questionnaires were assessed. Based on the students’ response the following opinions were noted. 25% of students believed brain death is equal to human death. 91% students responded that there is no treatment for brain dead patients. 62% of students accepted the concept of brain death. Religious and social reasons were the commonest reason for non-acceptance of brain death among the students. 97% of students believe that organ donation can save a life. 93% of students think that there is difference between organ transplantation from brain dead and cadaver. In students’ opinion, mutilation of the body was found to be the main reason for not accepting organ removal from brain dead patients.

## Conclusion

Religious and social factors were the major hindering factors for acceptance of brain death and organ donation. Knowledge of brain death and organ donation was lacking among medical students.

